# Fiberoptic endoscopic evaluation of swallowing in neurogenic oropharyngeal dysphagia in adults: Part 2 of a scoping review

**DOI:** 10.1007/s00405-026-10484-6

**Published:** 2026-08-03

**Authors:** Thaís Coelho Alves, Ana Maria Furkim, Antonio Schindler, Nicole Pizzorni, Suely Mayumi Motonaga Onofri, Roberta Gonçalves da Silva

**Affiliations:** 1 Dysphagia Research Rehabilitation Center; Speech, Language and Hearing Sciences Department, São Paulo State University (UNESP), Marília, São Paulo, Brazil, Marília, São Paulo Brazil; 2 Dysphagia Research, Teaching, and Extension Laboratory; Speech, Language and Hearing Sciences Department, Federal University of Santa Catarina (UFSC), Florianópolis, Brazil; 3 Department of Biomedical and Clinical Sciences, University of Milan, Milan, Italy

**Keywords:** Deglutition, Deglutition disorders, Endoscopy, Dysphagia, Review

## Abstract

**Purpose:**

The main objective of this scoping review was to describe the qualitative and quantitative parameters of fiberoptic endoscopic evaluation of swallowing (FEES) protocols, including their operational definitions and scales used in adults with neurogenic oropharyngeal dysphagia.

**Methods:**

Six databases (Wiley Cochrane Library, MEDLINE, EMBASE, CINAHL, PsycINFO, and LILACS) were extensively searched, following the Joanna Briggs Institute and PRISMA-ScR guidelines, without restriction on study design, publication date, or language. Two blind independent raters selected, reviewed, and extracted all content according to preestablished exclusion criteria. A third rater resolved any divergences between them.

**Results:**

Altogether, 6,033 abstracts were screened, leading to the inclusion of 115 publications. Substantial heterogeneity was observed in the terminology and operational definitions used to describe the qualitative parameters. Additionally, 20 different temporal quantitative parameters were identified, although only half of them were accompanied by operational definitions. Diverse classification methods were also reported, with author-defined measures being more common than validated scales.

**Conclusion:**

The parameters derived from FEES in adults with oropharyngeal dysphagia are not uniformly defined or analyzed.

## Introduction

Fiberoptic endoscopic evaluation of swallowing (FEES), first introduced in 1988 [1], has become an essential instrumental method for the assessment of oropharyngeal dysphagia, with numerous studies expanding its application across different clinical conditions, particularly in neurological disorders [2–5]. However, only more recently have the execution and interpretation of this examination gained greater attention, due to the lack of consensus regarding its diagnostic process and contributions to clinical decision-making [6].

Despite its widespread adoption in both clinical practice and research, important gaps remain in the literature. These gaps are particularly evident in the limited number of studies that clearly describe FEES protocols, raising concerns about the standardization of definitions and measurement methods [7]. In this context, this scoping review was structured into two complementary manuscripts, guided by previously defined research questions in adults with neurogenic oropharyngeal dysphagia: (1) what are the characteristics of the professionals responsible for performing and analyzing FEES; (2) what are the components of the protocols used; and (3) what qualitative pharyngeal parameters and temporal quantitative measures are analyzed using FEES.

The first manuscript (Part I) addressed the procedural aspects of FEES, including examiner characteristics and protocol components. Its findings revealed substantial heterogeneity in how FEES is conducted, raising important concerns regarding methodological standardization [8]. Considering these findings, the present manuscript (Part II) addresses a distinct but closely related dimension: the way swallowing outcomes are named, defined, and quantified in FEES. While Part I explored how the examination is performed, Part II examines how swallowing outcomes are conceptualized and measured.

This distinction is critical, as variability in parameter definitions, nomenclature, and measurement approaches may directly impact the interpretation of findings, the comparability between studies, and the development of evidence-based clinical protocols. In particular, the lack of consensus regarding operational definitions and the use of heterogeneous or non-validated scales represent key challenges for advancing the field.

Therefore, this second manuscript aimed to describe the qualitative and quantitative FEES parameters, including their operational definitions and classification methods, in adults with neurogenic oropharyngeal dysphagia.

## Methods

This scoping review followed the guidelines of the Joanna Briggs Institute (JBI) and the recommendations of the Preferred Reporting Items for Systematic Reviews and Meta-Analyses – Extension for Scoping Reviews (PRISMA-ScR). As this manuscript represents Part II of a two-part scoping review, full methodological details, including search strategy, eligibility criteria, and study selection process, are described in Part I of this review [8]. Briefly, six databases (Wiley Cochrane Library, MEDLINE, EMBASE, CINAHL, PsycINFO, and LILACS) were systematically searched. No temporal restrictions were applied to the search strategy, and studies were not restricted by publication year, study design, or language. Study selection and data extraction were independently performed by two blinded reviewers, with disagreements resolved by a third reviewer.

For the purposes of Part II, data extraction was specifically restricted to qualitative pharyngeal parameters, temporal quantitative measurements, their operational definitions, and the classification methods (scales) used in FEES. Extracted data were descriptively analyzed and organized according to parameter type and definition. As expected for a scoping review, no risk of bias assessment was performed.

## Results

### Literature

The study selection process followed the PRISMA-ScR guidelines and is described in detail in Part I [8]. A total of 6,033 records were identified across the databases, with 5,671 remaining after duplicate removal. Following title and abstract screening, 515 articles were selected for full-text assessment. After applying the eligibility criteria, 115 studies were included in this review. The complete list of included studies has been previously reported in Part I of this review [8].

### Parameters

Table 1 shows the distribution of articles according to qualitative and quantitative temporal parameters analyzed in FEES.

**Table 1:** Distribution of articles according to qualitative and quantitative temporal parameters analyzed in the fiberoptic endoscopic evaluation of swallowing (FEES)

	*n* (%)
*Qualitative parameters*	
Not performed/Performed	1 (0.9) / 114 (99.1)
Defined	40 (35.1)
Not defined	61 (53.5)
Not all parameters defined	13 (11.4)
*Qualitative parameters*	
Not performed/Performed	104 (90.4) / 11 (9.6)
Defined	7 (6.1)
Not defined	4 (3.5)

Of the 115 studies included, 114 (99.1%) analyzed qualitative parameters, and only 11 (9.6%) analyzed temporal quantitative measures. However, only 35.1% of those that evaluated qualitative parameters described their operational definitions. In the case of temporal quantitative parameters, this definition was even less frequent: only seven (6.1%) articles described all the parameters used, while four (3.5%) did not define any.

**Fig. 1:**
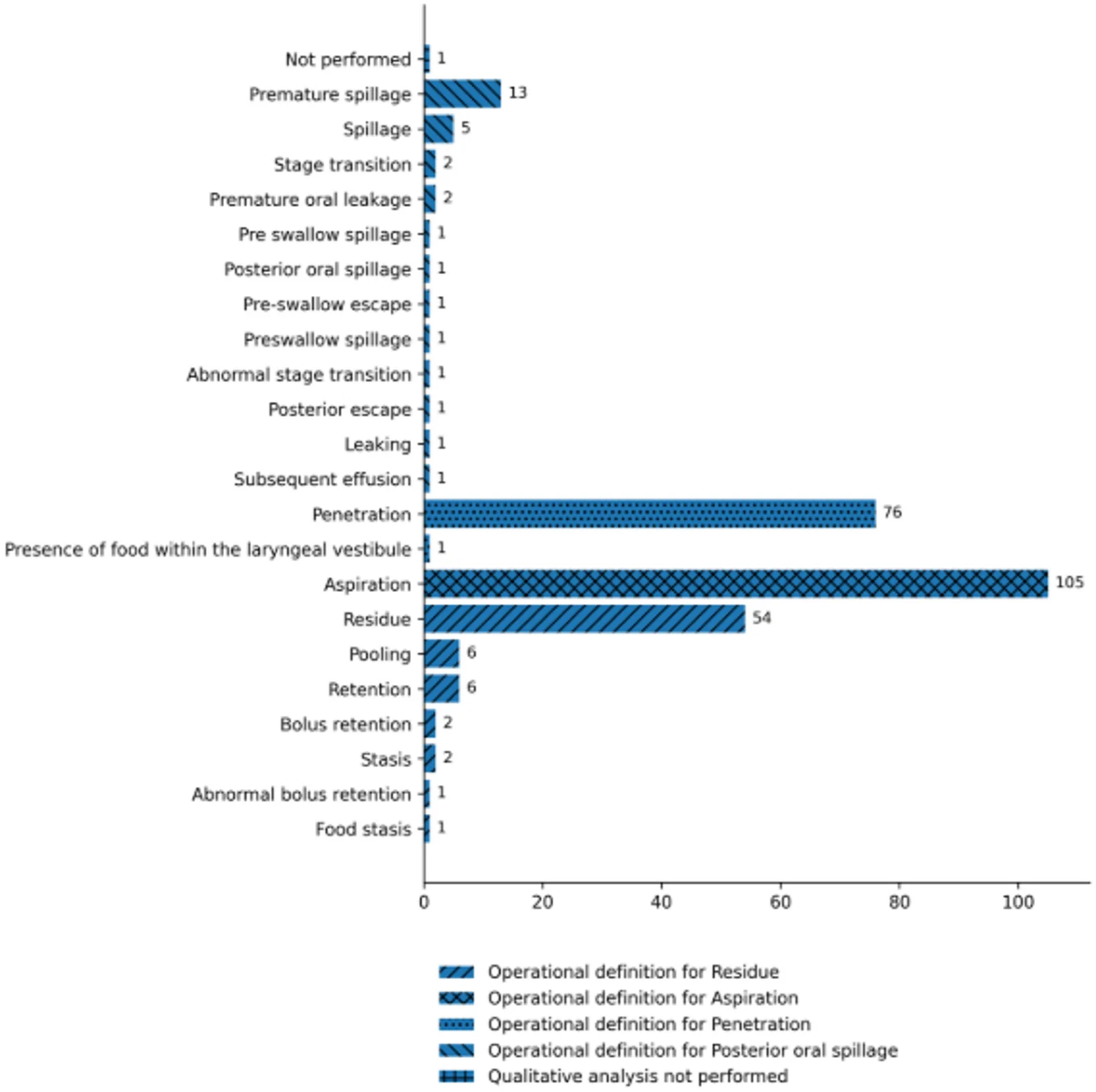
Nomenclatures and distributions of the operational definitions of posterior oral spillage, penetration, aspiration, and pharyngeal residue

Regarding the qualitative parameters analyzed in FEES, Fig. 1 shows the nomenclatures and distributions of the operational definitions of posterior oral spillage, penetration, aspiration, and residue found when extracting data from the 115 articles.

The studies used different nomenclatures for the operational definitions of posterior oral spillage, penetration, aspiration, and pharyngeal residue. Twelve different terms were identified for posterior oral spillage, with premature spillage being the most recurrent, mentioned in 13 articles. Regarding penetration, two terminological variations were found, with penetration appearing in 76 publications. As for pharyngeal residue, seven different nomenclatures were found, with residue being the most prominent, present in 54 studies. Finally, aspiration was referred to only with this nomenclature, used in 105 articles.

**Fig. 2:**
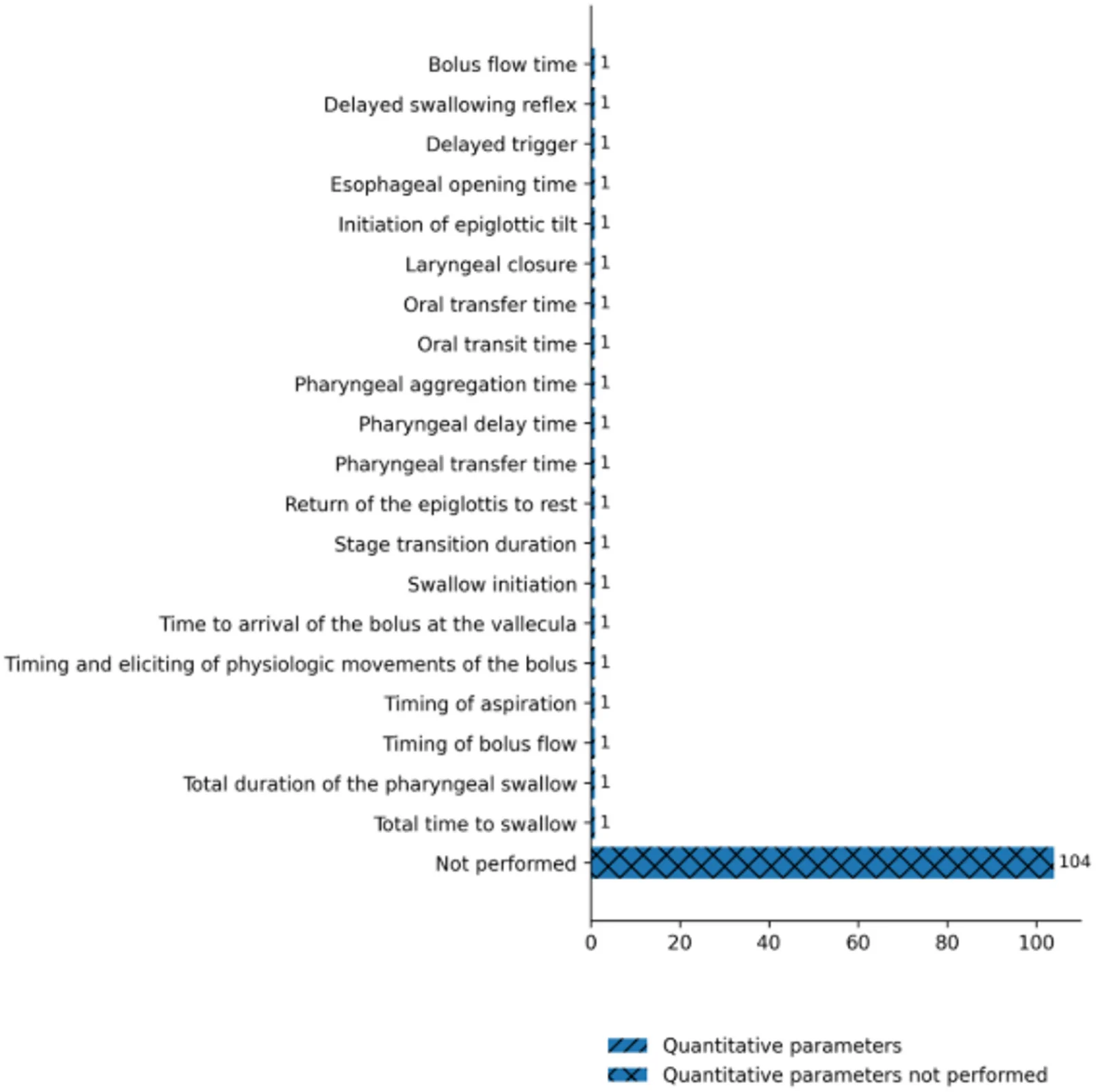
Distribution of temporal quantitative parameters analyzed by FEES

Figure 2 shows the temporal quantitative parameters analyzed by FEES that the review found, as well as their distributions. The review found 20 different parameters, each of them mentioned only once.

Furthermore, Table 2 shows the different definitions found for the operational definition of posterior oral spillage.

**Table 2:** Definitions found for the operational definition of posterior oral spillage

Definition of Posterior Oral Spillage	*n* (%)
*Defined by the depth of bolus flow to at least the vallecula prior to the pharyngeal swallow*	2 (1.7)
*Defined as the presence of the bolus at the hypopharynx (pyriform sinus) for more than 2 s before beginning the pharyngeal stage of swallowing*	2 (1.7)
*Defined as a bolus coursing into the hypopharynx more than 1 s before a swallowing response occurs*	1 (0.9)
*Defined as a swallowing stage*,* meaning before the pharyngeal swallow was initiated*	1 (0.9)
*Defined as the premature passage of food from the oral cavity to the pharyngeal cavity*	1 (0.9)
*Defined as material that enters the hypopharynx unintentionally from the oral cavity before initiating the pharyngeal swallow*	1 (0.9)
*Defined by the depth of bolus flow to the pyriform sinuses or bolus stasis in the vallecula or pyriform sinuses prior to the pharyngeal swallow*	1 (0.9)
*Defined as the head of the bolus entering the hypopharynx for more than 1 s before the swallowing response occurred*	1 (0.9)
*Defined as the progression of the food to the hypopharynx during the oral preparatory phase or after the pharyngeal phase of swallowing*,* while food residue was present in the mouth*	1 (0.9)

Nine different definitions were found for the operational definition of posterior oral spillage, with two definitions cited in two articles each.

Table 3 indicates the different definitions found for the operational definition of penetration.

**Table 3:** Definitions found for the operational definition of penetration

Definition of Penetration	*n* (%)
*Defined as any material entering the laryngeal vestibule but remaining at or above the level of the vocal cords*	5 (4.3)
*Defined as food material entering the laryngeal vestibule but not passing below the true vocal cords*	4 (3.5)
*Defined as material spilling into the laryngeal vestibule but not passing below the vocal cords*	2 (1.7)
*Defined as contrasted food seen in the laryngeal vestibule region above the vocal folds*	2 (1.7)
*Defined as any swallowed material entering the endolarynx but not below the vocal folds*	2 (1.7)
*Defined as material in the laryngeal vestibule but not passing below the level of the true vocal folds either before or after the pharyngeal swallow*	2 (1.7)
*Defined as material entering the larynx*	1 (0.9)
*Defined as material entering the airway either above or to the level of the vocal folds without entering the trachea and either ejected or not ejected*	1 (0.9)
*Defined as the presence of contrast material in the laryngeal vestibule*,* above the level of the vocal cords*	1 (0.9)
*Defined as the entrance of material into the laryngeal vestibule*	1 (0.9)
*Defined as the degree of intrusion into the laryngeal inlet without passing through the true vocal folds*	1 (0.9)
*Defined as the progression of the food to the glottis*,* without going beyond it*	1 (0.9)

The review found 12 different definitions for the operational definition of penetration; one of them was cited in five articles.

Table 4 shows the different definitions found for the operational definition of aspiration.

**Table 4:** Definitions found for the operational definition of aspiration

Definition of Aspiration	*n* (%)
*Defined as food material entering the airway below the true vocal cords*	17 (14.6)
*Defined as the passage of material below the vocal cords*	6 (5.2)
*Defined as material falling below the glottis*	2 (1.7)
*Defined as a bolus passed below the vocal cords*	1 (0.9)
*Defined as contrasted food seen on and/or below the free edges of the vocal folds*	1 (0.9)
*Defined as material passing below the vocal folds and either ejected or not ejected*	1 (0.9)
*Defined as the presence of contrast material at or below the upper edge of the vocal cords*	1 (0.9)
*Defined as any material passing below the vocal folds*,* thereby entering the trachea*	1 (0.9)
*Defined as material below the level of the true vocal folds*,* either before or after the pharyngeal swallow*	1 (0.9)
*Defined as the passage of material through the vocal folds into the trachea*	1 (0.9)
*Defined as food going beyond the level of the true vocal cords*,* up to the trachea*	1 (0.9)
*Defined as the passage of material under the level of the true vocal folds*	1 (0.9)
*Defined as a meal intrusion into the trachea seen after swallowing*	1 (0.9)
*Defined as the progression of the food below the vocal folds*	1 (0.9)

This table demonstrates that 14 different definitions were found for the operational definition of aspiration; one of them was cited in 17 articles.

Table 5 shows the different definitions found for the operational definition of residue.

**Table 5:** Definitions found for the operational definition of residue

Definition of Residue	*n* (%)
*Defined as retention of > 15% of a given material in the valleculae or the pyriform sinuses*	1 (0.9)
*Defined as material that more than coats the pharyngeal walls after swallowing*	1 (0.9)
*Defined as the material remaining in the pharynx after swallowing*	1 (0.9)
*Defined as residue in the pyriform sinuses or valleculae*	1 (0.9)
*Defined as persistence of green material along the pharyngeal walls or within the pyriform sinuses or valleculae*	1 (0.9)
*Defined as pooling in the valleculae and pyriform sinuses after swallowing*	1 (0.9)
*Defined as retention of the entire given materials in the valleculae or pyriform sinuses after swallowing*	1 (0.9)
*Defined as material insufficiently cleared from the hypopharynx during swallowing and staying back after swallowing*	1 (0.9)
*Defined as the presence of some or the entire tablet in the oropharynx following a swallow*	1 (0.9)
*Defined as the depositing of food in the valleculae and pyriform sinus*	1 (0.9)
*Defined as the presence of material in the pharynx after swallowing*	1 (0.9)
*Defined as abnormal bolus retention in the vallecula or pyriform sinuses after the pharyngeal swallow*	1 (0.9)
*Defined as bolus retention in the vallecula or pyriform sinuses after the pharyngeal swallow*	1 (0.9)
*Defined as persistence of food in the pharyngeal walls*,* pyriform sinus*,* or valleculae after swallowing*	1 (0.9)
*Defined as the persistence of material along the pharyngeal walls or within the pyriform sinuses or valleculae after swallowing*	1 (0.9)
*Defined as the presence of residual food in the piriform recesses and valleculae after three spontaneous deglutition attempts*	1 (0.9)

The review found 16 different definitions for the operational definition of residue; each definition was cited in a single article.

Table 6 shows the definitions in English of the temporal quantitative parameters found.

**Table 6:** Definitions of the temporal quantitative parameters found

Temporal quantitative parameters	*n* (%)
*Delayed trigger*: *Defined as more than 0.5 s after bolus pharyngeal entrance*	1 (0.9)
*Bolus flow time*: *Defined as the measurement from the time the bolus is seen in the hypopharynx until it triggers the swallowing reflex*	1 (0.9)
*Total duration of the pharyngeal swallow*: *Defined as the time interval between the beginning of epiglottal inversion and the definite epiglottal return to the resting position. The end of the pharyngeal swallow was defined as the time point when the other pharyngeal structures (i.e.*,* the pharyngeal walls) had returned to their resting position. The total duration of the pharyngeal swallow was calculated as the average sum of the above*	1 (0.9)
*Stage transition duration*: *First frame of bolus arrival when the endoscope was in home position to the first complete white-out frame*	1 (0.9)
*Swallow initiation*: *Defined as the timing of a “white-out” during swallowing in image of fiberoptic endoscopic evaluation of swallowing*	1 (0.9)
*Pharyngeal aggregation time*: *Defined as the interval from when the leading edge of any component (liquid or solid) of the food reaches the base of the epiglottis until the first swallow*	1 (0.9)
*Oral transfer time*: *Defined from oral-velar opening (velum elevation from the dorsum of the tongue) to oral-velar closure*	1 (0.9)
*Pharyngeal transfer time*: *Defined as bolus passage from pharynx to esophagus*	1 (0.9)
*Esophageal opening time*: *Defined as the interval from opening until closure of the upper esophageal sphincter*	1 (0.9)
*Total time to swallow*: *Defined as the interval from the placement of the tablet on the tongue until clearance from the oropharynx on the endoscopic record*	1 (0.9)

The review found only 10 definitions, each one cited in only one article.

Table 7 presents the classification methods (scales) for saliva/secretion residue, residue after swallowing, penetration/aspiration, other analyses, and temporal quantitative analysis.

**Table 7:** Distribution of classification methods (scales) described in the articles included in this scoping review

	*n* (%)
Own measurements (dichotomous or other methods)	57 (49.6)
Not described	2 (1.7)
Saliva/secretion residue	
* Murray Scale*	8 (6.8)
* Basal Secretion Scale of Langmore*	2 (1.7)
* New Zealand Secretion Scale*	1 (0.9)
* Secretion Severity Rating Scale*	1 (0.9)
* 5-point secretion scale*	1 (0.9)
Residue after swallowing	
* Pooling Score Scale*	6 (5.2)
* Yale Pharyngeal Residue Severity Rating Scale*	5 (4.3)
* Residue rating scale*	2 (1.7)
* Scale for post-swallow pyriform sinus pooling*	1 (0.9)
* Scale for post-swallow vallecular pooling*	1 (0.9)
* Scale for residue severity*	1 (0.9)
Penetration/Aspiration	
* Penetration aspiration scale*	39 (33.5)
* Depth of bolus penetration*	1 (0.9)
* Scale for penetration-aspiration*	1 (0.9)
* Airway invasion*	1 (0.9)
Other analyses by scale	
* Fiberoptic endoscopic dysphagia severity scale*	3 (2.6)
* Endoscopic severity of dysphagia scale*	1 (0.9)
* Hyodo-Komagane score*	1 (0.9)
* Pooling sensation-collaboration-age score*	1 (0.9)
* Scoring of FEES examinations*	1 (0.9)
Quantitative analysis	
* Scale for delayed initiation of the pharyngeal reflex*	1 (0.9)

It was found that own measurements (dichotomous methods or others) were the most frequently mentioned, followed by the PAS.

## Discussion

This review highlights substantial variability in the definition, nomenclature, and measurement of FEES-derived swallowing parameters in adults with neurogenic oropharyngeal dysphagia. Despite the widespread use of FEES in both clinical practice and research, important gaps and inconsistencies persist in how swallowing outcomes are defined and operationalized [4, 7, 9–12]. Across the 115 included studies, considerable heterogeneity was observed in the terminology, operational definitions, classification methods, and quantitative measurements used in FEES.

The findings of the present review, together with those of previous studies, reflect a growing movement within the FEES literature toward better characterization, standardization, and validation of the parameters used to describe swallowing findings. In this context, Swan et al. [13] identified important limitations in the evidence supporting the validity and reliability of visuoperceptual measures used in FEES and videofluoroscopy, highlighting the need for more robust and standardized assessment frameworks. More recently, Cordier et al. [14] conducted an international Delphi study, establishing consensus-based definitions, items, and operationalizations for FEES assessment. Collectively, these initiatives complement one another and demonstrate the field’s current concern with reducing heterogeneity related to nomenclature, operational definitions, validity evidence, classification methods, and interpretation of FEES findings.

The 115 articles included in this scoping review widely reported qualitative swallowing parameters, such as posterior oral spillage, pharyngeal residue, laryngeal penetration, and aspiration. However, despite the frequency with which these outcomes are mentioned, only aspiration had relatively consistent operational definitions across studies. In contrast, the concept of parameters such as posterior oral spillage and pharyngeal residue showed considerable heterogeneity, demonstrating that their exact understanding in FEES remains unclear and inconsistently applied in the literature. This variability suggests that even widely recognized clinical findings lack stable definitions regarding their presence and clinical interpretation.

Despite the extensive reporting of qualitative swallowing parameters, few studies have systematically explored the temporal analysis of swallowing using FEES. A possible explanation for this gap may be the technical and methodological challenges, including limitations in video quality, the need for frame-by-frame or slow-motion analysis, and the requirement for specific analytical tools or software [5, 15, 16].

The present review identified three main patterns in how studies addressed FEES parameters: (1) studies that provided complete operational definitions; (2) studies that did not define the parameters analyzed; and (3) studies that defined only part of the parameters. These findings reinforce the lack of consistency in how FEES parameters are described and reported across studies.

Furthermore, extensive terminological variability was observed in association with the operational definitions used, without a consolidated consensus among authors. The review identified 12 nomenclatures and nine distinct definitions for posterior oral spillage; two nomenclatures and 12 definitions for penetration; seven nomenclatures and 16 definitions for pharyngeal residue; and, despite the uniform terminology of aspiration, 14 different operational definitions were identified. These findings indicate that, even when there is terminological agreement, significant conceptual variability persists in the way phenomena are operationalized and interpreted, compromising the comparability between studies and the synthesis of evidence.

In addition to qualitative parameters, 20 distinct temporal quantitative parameters were identified, of which only half were accompanied by explicit operational definitions. This lack of clarity in temporal parameter description further limits the reproducibility of FEES-based analyses and restricts the integration of findings across studies.

In recent years, there has been a movement toward the development of more consensual and validated FEES protocols, together with consensus-building initiatives such as Delphi studies, as well as studies aimed at promoting good practices, patient safety, and greater scientific reproducibility [14, 17]. In this context, the findings of this review reinforce the importance of critically examining which operational definitions, nomenclatures, and measurement approaches should be adopted in future protocols [18].

The variability observed also extends to the classification methods used to interpret FEES findings. Author-developed measures, including dichotomous classifications and non-validated scoring systems, were frequently reported, followed by the use of standardized tools such as the Penetration-Aspiration Scale (PAS) [19, 20]. However, Langmore [4] emphasizes that aspiration should not be interpreted as an isolated event nor exclusively based on PAS, highlighting the importance of considering the time of occurrence for understanding the mechanism involved and defining appropriate therapeutic interventions. Moreover, the systematic review by Swan et al. [13] identified relevant gaps in the evidence of validity and reliability of the scales used in FEES, indicating a lack of robust scientific support for recommending an individual measure as a validated standard.

Importantly, these findings complement those described in Part I of this review [8], which focused on the procedural aspects of FEES. While Part I demonstrated substantial variability in how the examination is performed, the present manuscript reveals that this heterogeneity extends critically to how swallowing outcomes are defined, named, and measured. Together, these findings suggest that methodological inconsistency in FEES encompasses both procedural and analysis of outcomes, reinforcing the need for integrated standardization.

Despite the methodological rigor adopted, some limitations should be considered, such as the exclusion of grey literature and pediatric populations or other groups besides adults with oropharyngeal dysphagia. Even so, the findings provide relevant support for the development of standardized FEES protocols applicable to adults with neurogenic oropharyngeal dysphagia.

Overall, this review highlights that advancing the field of FEES requires not only improvements in how the examination is performed but, critically, in how its findings are defined, categorized, and measured. This review reinforces the need for more consensual and clear FEES protocols that integrally address examination procedure and parameter analysis, aiming for greater methodological reliability, study comparability, and clinical impact.

## Conclusion

The findings of this scoping review demonstrate that FEES parameters applied to adults with neurogenic oropharyngeal dysphagia lack uniformity. Wide variability was observed not only in the nomenclatures used but also in the operational definitions of the qualitative and quantitative temporal parameters, as well as in the scales used for their analysis.

This heterogeneity compromises comparability across studies, hinders the synthesis of evidence, and limits the reproducibility of findings, with potential implications for clinical interpretation. Thus, the results of this review highlight the need for efforts directed towards the terminological and operational standardization of FEES parameters to strengthen the methodological quality of future research, favoring the translation of scientific knowledge into clinical practice.
